# The distress of psychological adaptation in nutritional management among people after esophagectomy: an interpretative phenomenological study

**DOI:** 10.3389/fnut.2026.1720415

**Published:** 2026-02-27

**Authors:** Chang Ying Liu, Qing Zhang, Chun Yan Zhang, Ying Yang, Yun Yun Chen

**Affiliations:** The Affiliated Huaian No. 1 People's Hospital of Nanjing Medical University, Huaian, Jiangsu, China

**Keywords:** esophageal cancer, mental health, nutritional management, nutrition-related symptoms, qualitative research, weight restoration

## Abstract

**Background:**

Patients who undergo esophagectomy frequently encounter long-term nutrition impact symptoms (NISs) and dietary challenges resulting from anatomical and functional alterations. Although these issues adversely affect mental health, the underlying psychological mechanisms remain poorly understood. This study aimed to explore the psychological experiences of post-esophagectomy people diagnosed with esophageal cancer regarding nutritional management to elucidate the impact of nutritional challenges on mental health.

**Methods:**

A purposive sample of 16 who had undergone esophagectomy for esophageal cancer was recruited. Data were collected via semi-structured, in-depth interviews and analyzed using Interpretative Phenomenological Analysis (IPA). The analysis followed the hermeneutic circle principle through iterative coding, theme development, and the synthesis of cross-case patterns.

**Results:**

Three principal themes emerged: (1) cognitive and behavioral adaptation to gastrointestinal symptoms; (2) adaptive challenges and identity reconstruction in family dietary contexts; and (3) survival significance and social pressures in nutritional management. Collectively, these factors reinforced the “cancer patient” identity and impeded psychological recovery.

**Conclusion:**

This study revealed that persistent NISs, unmet recovery expectations, and role-identity conflicts contribute to anxiety, helplessness, and confusion regarding health identity. These findings emphasized the necessity of patient-centered nutritional interventions that address symptom-related cognitive anxiety, facilitate familial support for dietary adaptation, and balance disease risk management with the enjoyment of food. These insights provide a theoretical foundation for developing personalized, integrated nutritional and psychological support programs to enhance the mental health and quality of life of esophageal cancer survivors.

## 1 Introduction

Esophageal cancer is a major malignancy of the upper digestive tract, ranking as the seventh most frequently diagnosed cancer and the sixth leading cause of cancer-related mortality worldwide (1). Surgical resection remains the primary treatment for localized esophageal cancer, involving the excision of substantial esophageal segments and reconstruction using a gastric conduit (2). However, the necessary vagotomy and the anatomical alterations following esophagectomy with gastric reconstruction disrupt gastric motility, pancreatic enzyme secretion, bile salt absorption, and gastrointestinal hormone regulation (3, 4). Furthermore, the loss of parietal cells and intrinsic factor impairs vitamin B12 absorption. Altered gastrointestinal physiology also hinders iron and calcium uptake, leading to micronutrient deficiencies (3, 5). These pathophysiological changes result in long-term nutrition impact symptoms (NISs), which including dysphagia, early satiety, dumping syndrome, anorexia, reflux, nausea, vomiting, diarrhea, dietary restrictions, and malabsorption. Collectively, these symptoms adversely affect physical, emotional, and social wellbeing, thereby compromising their overall quality of life (3, 4, 6–8).

Nutrition management and symptom relief are recognized as integral components of cancer survivorship (9). Current guidelines prioritize strategies such as regular malnutrition screening, preoperative and postoperative nutrition support, the consideration of dietary components, and the implementation of nutrition care within a multidisciplinary team to optimize outcomes (10). Previous research has investigated the nutritional challenges, coping strategies, and unmet information needs among populations facing severe nutritional complications, particularly survivors of esophageal and gastric cancer (11, 12). Dietary modification is not only a direct consequence of esophagectomy but also a pivotal strategy for managing postoperative symptoms (12). However, these adjustments significantly burden daily life, meal preparation becomes excessively time-consuming, and social interactions are often curtailed (11, 12). Notably, these diet-related symptoms and adaptation challenges are rarely transient and may persist for years (13). A retrospective study reported that, even 30 months after surgery, only 72.8% of patients with esophageal cancer had returned to their pre-surgical eating frequency, and reflux prevalence remained as high as 38.4%, accompanied by an average weight loss of 10% (14).

The association between the nutritional and psychological states of cancer survivors has garnered increasing attention, with existing research primarily focusing on the biological mechanisms linking dietary components to mental health (15). However, diet encompasses more than physiological necessity; it serves as a vital framework for structuring daily life and fostering a sense of collective belonging. This function is influenced by multifactorial elements including life experiences, beliefs and expectations, social interactions, and the food choice environment (16–18). For post-esophagectomy patients, altered dietary patterns can lead to a state of ‘embodied disorientation,’ wherein these patterns pose a persistent threat to dignity, functional roles, social relationships, and daily activities (12). Consequently, nutritional symptoms and management challenges may hinder the transition from a “patient” to “healthy individual” (19), which may contribute to psychological distress, such as anxiety and depression (20).

This study hypothesized that the mental health of esophageal cancer survivors following esophagectomy is influenced not only by persistent symptoms but also by nutrition-related subjective cognition, behavioral regulation, and sociocultural factors. Grounded in cognitive theory and phenomenological perspectives, a detailed examination into the psychological adaptation to nutritional changes in this population can help elucidate the pathways linking nutritional challenges to mental health outcomes. Accordingly, this study examined the explored the lived experience of these patients regarding nutritional management. It aimed to reveal adaptation processes concerning gastrointestinal symptoms and dietary behavior within the contexts of internal cognitive frameworks and external social interactions. By analyzing these pathways, this research seek to provide a theoretical foundation and practical insights for developing integrated, personalized interventions that address both nutritional and psychological needs.

## 2 Methods

### 2.1 Study design

This study adopted Interpretive Phenomenological Analysis (IPA) (21) as its methodological framework. IPA examines how individuals make sense of major life experiences within specific contexts, aiming to provide a detailed interpretation of the personal meaning attributed to these experiences. This approach aligns well with the study’s objective: to investigate how postoperative esophageal cancer patients psychologically adapt to gastrointestinal symptoms and altered dietary behaviors.

### 2.2 Ethics

Ethics approval was obtained from the authors’ The Affiliated Huaian No.1 People’s Hospital of Nanjing Medical University on 3 July 2024 (Approval Code: MR-32-25-031754). Prior to data collection, all participants were provided with an information sheet and gave written informed consent. To ensure confidentiality, all transcribed data was anonymized. Each participant was assigned a unique identification code (e.g., P1), which is used to identify their quotations throughout the manuscript. Consent forms and audio recordings were securely stored in a locked cabinet within the principal investigator’s office, accessible solely to the research team.

### 2.3 Sample and setting

From April to August 2025, participants were selected from The Affiliated Huaian No.1 People’s Hospital of Nanjing Medical University using purposive sampling, with sample size determined by data saturation. Inclusion criteria were: (1) pathologically confirmed as esophageal cancer treated with radical esophagectomy; (2) 3 to 12 months post-surgery and discharged from the surgical ward; (3) age ≥18 years; (4) adequate communication abilities; and (5) provision of written informed consent. Exclusion criteria included: receiving tube feeding or parenteral nutrition; presence of severe complications (e.g., cardiovascular disease, other malignancies); significant cognitive impairment or psychiatric disorders; receipt of palliative care, or lack of disease awareness. Demographic information was collected using a structured questionnaire. Data collection and preliminary analysis proceeded iteratively. Recruitment continued until thematic saturation was reached, defined as the point where no new insights regarding psychological adaptation, identity reconstruction, or nutritional meaning-making emerged from two consecutive interviews. Saturation was confirmed through continuous team discussions, where new data were compared with established themes until conceptual repetition was evident. This was achieved with 16 participants. Of the19 eligible survivors invited, 16 consented and completed the study (3 declined). Table 1 present the characteristics of the 16 participants, whose ages ranged from 58 to 75 years (mean = 64.6 years).

**Table 1:** Demographic and clinical characteristics of patients.

ID	Gender	Age (yr)	Education level	Occupation	Time since surgery	Current treatment	Living situation
P1	Male	58	Bachelor’s	Ret. Mil.	3 months	Chemotherapy	A
P2	Male	65	Middle school	Farmer	5 months	Chemotherapy	B
P3	Female	62	High school	Homemaker	6 months	None	B
P4	Male	67	Middle school	Ret. Mil.	4 months	Chemotherapy	A
P5	Male	62	High school	Self-employed	12 months	None	A
P6	Female	66	Middle school	Homemaker	6 months	None	B
P7	Female	65	Primary school	Farmer	4 months	Chemotherapy	B
P8	Male	72	Middle school	Retired	10 months	None	B
P9	Female	58	High school	Self-employed	6 months	None	A
P10	Male	67	Middle school	Retired	12 months	None	B
P11	Female	65	Middle school	Retired	10 months	None	A
P12	Male	59	High school	Self-employed	8 months	None	A
P13	Male	63	Middle school	Retired	5 months	Chemotherapy	A
P14	Female	66	High school	Retired	10 months	None	B
P15	Male	65	High school	Retired	12 months	None	A
P16	Male	75	Primary school	Retired	8 months	None	B

Ret. Mil., retired military; A, living with spouse; B, living with family members (including intergenerational family).

### 2.4 Data collection

Data were collected using semi-structured, in-depth interviews. The research team was exclusively female. Interviews were conducted by authors Liu CY and Zhang Q, both of whom hold Master of Nursing degrees, have received specialized training in qualitative methods, and possess extensive experience in conducting and publishing qualitative research. The interview guide was developed based on the research aims and relevant literature to explore participants’ experiences, perceptions, and coping processes. The guide covered four domains: (1) Symptom (e.g., “What uncomfortable feelings have you experienced regarding eating? What do these feelings mean to you?” “How do you manage these symptoms?); (2) eating routine (e.g., “How has your daily eating routine changed since surgery?”; “What does these changes mean to you?”; “How do you regulate your food intake?”); (3) nutrition-related family interactions (e.g., “What are your current dining arrangements with your family?”; “What are your thoughts on this dynamic?; “How do you navigate this situation?”); and (4) weight (e.g., “How has your weight changed since the surgery?”; “What does these changes mean to you”; “How are you adjusting to these changes?”). The interview guide was designed to be open-ended, allowing participants to direct the narrative flow, with probing questions adapted dynamically by the interviewer. Interviews were conducted in quiet, comfortable settings by the first author (Liu CY).

A limited professional relationship existed with some participants, as the interviewer had previously provided nursing care during their clinical treatment. To mitigate power imbalances, a de-medicalization strategy (applied to setting, attire, and language) was implemented. Reflexivity was maintained through the use of reflective journals and member checking to manage potential biases. Field notes were recorded during interviews, and clarifications were sought as needed. Interviews concluded when participants indicated they had no further information to share. Eleven interviews were conducted face-to-face in a designated hospital interview room, while five were conducted via telephone. All interviews were conducted in the participants’ local dialect and were audio-recorded in full. The interviews lasted between 20 and 75 min, with a mean duration of approximately 43 min. No repeat interviews were performed. The study adhered to the Consolidated Criteria for Reporting Qualitative Research (COREQ) checklist (Supplementary File 1).

### 2.5 Data analysis

Microsoft Word 2021 was utilized to manage and code interview transcripts. Audio recordings were transcribed verbatim within 24 h of each interview, and transcripts were supplemented with field notes to capture non-verbal cues. To ensure accuracy and credibility, the transcripts were reviewed and cross-checked by authors Liu CY, Zhang Q and Zhang CY. Data analysis was conducted by authors Liu CY and Zhang Q in accordance with the hermeneutic circle principle of the IPA framework. The process as follows: (1) Immersion and Annotation: analysts immersed themselves in the dataset by reading the transcripts and re-listening to audio recordings at least twice. During this phase, they annotated the text to identify phenomenologically salient content, including distinct linguistic features (e.g., metaphors, repetitions) and emotional expressions, while recording their own reflective notes; (2) developing Emergent Themes: Analytical notes were transformed into emergent themes. This involved formulating concise phrase at a slightly higher level of abstraction while ensuring the themes remained grounded in the participants’ narratives; (3) clustering: Emergent themes were clustered based on conceptual relationships (e.g., similarities or contradictions), and descriptive labels were assigned to each cluster; (4) cross-case Analysis: thematic clusters were compared across all 16 participants to identify convergent and divergent patterns. During the refinement process, a theme was retained only if it was supported by excerpts from at least three participants. Discrepancies were resolved through collaborative review of the transcripts and coding structure until consensus was reached.

### 2.6 Researcher reflexivity

Interviews were conducted by the first author (Liu CY). Regarding data analysis, authors Liu CY and Zhang Q independently performed the initial coding and theme development. Throughout this process, each analyst maintained a reflective journal to document interpretive decisions and personal reflections. Structured research meetings were convened at critical junctures: following initial coding, after theme clustering, and prior to the finalization of superordinate themes. During these sessions, the analysts compared coded extracts, emergent themes, and reflective notes. Discrepancies were discussed in depth, with the third author (Chen YY) serving as a critical peer to challenge interpretations and ensure that all themes remained grounded in the participants’ narratives.

## 3 Result

Three principal themes emerged from the analysis, capturing the complex psychological experience of nutritional management among cancer patients following esophagectomy: (a) cognitive and behavioral adaptation to gastrointestinal symptoms; (b) adaptive challenges and identity reconstruction in family dietary contexts; and (c) survival significance and social pressures in nutritional management. These themes are underpinned by nine sub-themes, which provide an in-depth understanding of the patients’ cognitive, emotional, and behavioral adaptations. A structured coding tree detailing these themes and sub-themes is provided in Supplementary File 2.

### 3.1 Cognitive and behavioral adaptation to gastrointestinal symptoms

#### 3.1.1 Staged evolution of gastrointestinal symptoms cognition

Participants’ understanding of gastrointestinal symptoms evolved through distinct stages, paralleling the cognitive adjustments involved in adapting to their illness identity. Initially, symptoms were accepted as tolerable when interpreted as inevitable treatment outcome or when validated by medical professionals (e.g., physicians) as safe and transient.

*"Such a major surgery naturally causes discomfort… At first, I was worried, but the doctors saying it was perfectly normal, so I gradually got used to it," P1; "The doctors have already checked and said it's a normal reaction," P6.*

However, a significant shift in cognitive framing occurred following hospital discharge, an event that marked a symbolic transition in social roles. The persistence of symptoms beyond the anticipated recovery timeline constituted a violation of expectations. This discrepancy triggered a cognitive conflict between the expected return to health and the enduring reality of illness. Consequently, participants began to question their recovery progress, attributing ongoing symptoms to potential behavioral errors or physiological defects. This internalization fueled anxiety regarding functional abnormality, a contradiction made particularly difficult to reconcile by the intense desire to reclaim the identity of a “healthy individual.”

*"How am I still experiencing belching after four months?," P7; "How on earth could a normal recovery take so long!", P3*. *"I see my neighbor wanting to eat, but why don't I want to eat? Am I not recovering well?," P13; "…can't even eat, (I'm) useless," P2*.

#### 3.1.2 Uncontrolled trauma in symptom self-management

Based on their evolving understanding of symptoms, participants typically initiated control-oriented self-regulation strategies. They operated under the belief that symptom exacerbations had specific, identifiable causes and that the responsibility for identifying and mitigating these triggers lay with them. Given that post-operative gastrointestinal symptoms were highly sensitive to dietary and behavioral choices, participants engaged in “experiential analysis” to determine effective coping strategies. For the subset of participants who achieved effective symptom control, this success fostered an enhanced sense of control and self-efficacy, accompanied by reduced anxiety. These positive experiences facilitated a transition in self-perception from that of a ‘passive patient’ to an ‘active health manager.’

*"This time I ate a bit more and had reflux, so now I have a better sense of how to manage it…I'm in better condition than many others; they were rather pitiful." P1; "As long as I know how to handle it, it’s manageable," P4.*

Conversely, when iterative adjustments failed or symptoms remained unpredictable, this adaptive process was disrupted. The majority of participants struggled to implement effective coping strategies, resulting in profound psychological distress characterized by frustration, fear, and a deteriorating self-concept.

*“Well, I’ve taken the medication as prescribed, but I still cannot eat. I’m useless,” P2; “I vomit acid every day; I’m scared to eat, but also scared not to eat,” P13.*

Although physical rehabilitation gradually alleviated symptom severity, the persistent threat of recurrence generated a state of underlying psychological tension and anticipatory anxiety. This fear extended beyond specific physical sensations to question the reliability of the body itself, thereby hindering the integration of the postoperative physical state into a “new normal” and impeding the reconstruction of a healthy identity.

*"I still feel a bit worried when eating, afraid that reflux might happen again. If I could eat normally again—without having to worry anymore—it would mean I'm completely well," P12.*

#### 3.1.3 Dynamic testing of dietary and physiological boundaries

Because participants typically received generalized recovery guidance, they relied on personal experience and iterative trial-and-error to develop individualized dietary strategies. The drastic reduction in gastric capacity following surgery created an extremely narrow tolerance for food intake, making it challenging to gauge meal adequacy using familiar satiety cues. During early recovery, a conflict often arose between a strong desire to eat and limited physiological capacity, frequently resulting in gastroesophageal reflux caused by overeating.

*"I ate too much without feeling full," P12; "I can't eat even one extra bite; just one bite makes me throw up," P4.*

As participants recognized the direct correlation between intake volume and symptoms, they were compelled to adopt rigid portion controls, a strategy that often precluded the natural sensation of fullness.

*"I must use my small bowl; it's one bowl each time," P1; "I can't feel full, so I have to eat more smaller meals," P4.*

However, driven by perceived functional improvements and an intense desire to resume preoperative eating habits, participants persistently engaged in “boundary crossing.” The outcomes of these experiments continually reshaped their perceptions of recovery progress and the feasibility of reclaiming the identity of a “healthy individual.”

*"I definitely want to return to three meals a day, so I'll try eating a bit more every two days…I knew cancer wasn't catching, but I still preferred to keep my distance… Once I'm eating normally, I'll join my family at the table," P1; "I feel hungrier today, so I'll try an extra bite to see if it works…If you're eating normally again, that means you're all good," P3.*

#### 3.1.4 Negotiating dietary pleasure and perceived recurrence risk

Participants’ dietary decisions were profoundly shaped by sociocultural beliefs. Specifically, influenced by the Traditional Chinese Medicine (TCM) concept of *fawu* (foods believed to induce illness recurrence), participants classified certain nutrient-dense foods like lamb and fish as potential triggers for cancer recurrence.” Consequently, Participants proactively avoided these items to establish a sense of control amidst uncertainty. While unable to control the fundamental trajectory of the disease, adhering to these dietary taboos provided a form of “symbolic mastery” over perceived risks, thereby alleviating recurrence-related anxiety.

*"Better to believe it exists than to believe it doesn't," P15; "If we can avoid it, we should; it must have some benefit (in preventing cancer)," P5.*

Similarly, most participants felt compelled to abandon preferences for strong-flavored foods that were perceived as unhealthy. Within a cultural context that highly values culinary enjoyment as integral to quality of life, such abstinence resulted in a profound sense of deprivation. These trade-offs compounded practical and emotional burden of nutritional management, ultimately reinforcing the self-perception of being a “highly restricted patient.”

*"I definitely want to eat it, I'm craving it," P5; "I'm afraid I might give in and eat those foods, so I just don't dine with them. You know, ‘out of sight, out of mind,*’*" P4.*

### 3.2 Adaptive challenges and identity reconstruction in family dietary

#### 3.2.1 Self-imposed marginalization at shared meals

In adapting to their new dietary habits, participants had to undergo role reconstruction, transitioning from “regular diners” to “individuals with special dietary needs.” The marked disparity in their meals attracted heightened attention from family members, intensifying their self-identification as cancer patients. To avoid this focus, they voluntarily withdrew from shared family meals.

*"When eating together, everyone watches how much I eat," P1; "It’s more comfortable for them to eat without me," P3.*

Some participants, experiencing symptoms like hiccups and belching, feared disrupting the dining atmosphere for others. This concern reflected the internalization of lost bodily control as a stigmatizing trait, which exacerbated their sense of being a burden.

*"Even family members might dislike it when I hiccup," P2; "No matter what, it inevitably affects them," P7.*

Thus, opting for spatial isolation at mealtimes served as a strategy to manage the visibility of their illness and mitigate the risk of social judgment within the family. While this approach offered temporary relief from interaction anxiety, the physical segregation symbolically reinforced a sense of “otherness,” thereby impeding their reintegration into the family as equal members.

#### 3.2.2 The dilemma between familial expectations and personal capacity

Family dietary interactions involved a complex negotiation between expectations and reality. Participants actively controlled their food intake to avoid symptoms, whereas family members driven by recovery expectations and a lack of symptom awareness, often encourage increased consumption. Participants acknowledged this goodwill and internalized an emotional obligation to comply. However, the disparity between their physiological capacity and these external demands created a conflict between their physical needs and emotional responses.

*"My wife always wants me to eat more, and I eat to make her happy, but I feel unwell and can't handle it," P3;"*

This tension was acutely exacerbated during chemotherapy, a period often characterized by reduced appetite. When their food intake consistently fell short of family expectations, participants experienced being pressured to eat. This dynamic not only intensified their sense of powerlessness and eating-related anxiety but also triggered the internalization of physiological limitations as personal failures, ultimately eroding their self-worth.

*"I've already said I don't want to eat, but she keeps bringing food over, saying, ‘Just a little bit.’ I can't even eat; I’m useless," P2; "Family members mean well when they urge me to eat more, but It’s just hard to achieve. I know eating is important, and I'm also anxious," P13.*

#### 3.2.3 The symbolic meaning of dietary reintegration into family routines

For most participants, fulfilling specific roles within family dietary routines (e.g., minimizing food waste) had been a meaningful way to contribute to the family system. However, physiological limitations compelled them to withdraw from these functional roles. This withdrawal signaled loss of utility in the family’s daily functioning, thereby reinforcing their identity as patients.

*"I used to clear the plate by eating a few extra bites, but now I can’t manage a single bite more. I wanted to give it a try, but my wife stopped me, saying it wasn’t necessary for me," P4; "I truly couldn't eat more, so my family said they had no choice but to discard the food," P10.*

Therefore, participants viewed the realignment of their diet with family norms as a symbolic step toward identity normalization. The ability to share communal meals without dietary distinction signified an exit from the category of ‘patients requiring special care.’ This shift marked the initial reintegration into a normal social identity within the family.

*"Now it's nothing special; we all eat together, and I have the same meals as my family," P10; "We just eat together normally, and usually don't bring up the matter," P11.*

### 3.3 Survival significance and social pressures in nutritional management

#### 3.3.1 Adaptive challenge of weight recovery expectations and realistic outcomes

A minority of participants interpreted unintentional weight loss as a correction of a prior overweight state, successfully integrating this physical change into a healthier self-narrative.

*"I used to be a little fat, but now I've lost a little weight. I think it's just right, healthier than before," P9.*

However, the majority failed to achieve this integration. They remained entrenched in a cognitive schema wherein the ability to gain weight through adequate intake is viewed as an inherent capacity of a healthy body. Consequently, as symptoms and dietary patterns approached baseline levels, participants expected a corresponding recovery in weight. The failure of reality to meet this expectation precipitated cognitive conflict. A common coping strategy involved internal attribution, whereby participants ascribed the failure to regain weight to insufficient personal effort. By re-framing an uncontrollable physiological outcome as a personal failure, they constructed a coherent, albeit painful, explanatory framework for an otherwise inexplicable reality. However, this process simultaneously undermined their sense of self-efficacy.

*"I'm eating quite a lot now, but my weight just won't come back. What am I doing wrong?," P5; "I'm eating better than before, but my weight hasn't increased. I don't know what else to do," P8.*

Notably, even when provided with comprehensive medical explanations regarding metabolic alterations, these internal attribution and cognitive conflicts persisted. This persistence highlights the disparity between intellectual understanding and emotional acceptance, driving a continuous search for resolutions.

*"The doctor told me I would lose weight, but I still feel I need to try harder," P1; "I've seen some people regain a lot of weight, but no matter how much I eat, I can't. Does it mean my condition is more severe?," P10.*

#### 3.3.2 Emaciation as a visible disease stigma

Unintentional weight loss resulting in bodily emaciation served as a persistent and visible marker of illness, which exacerbated a negative body image.

*"I've become so thin that I look ill," P8; "You know why I wear so many layers? To appear a bit heavier," P5.*

Within Chinese sociocultural frameworks, an emaciated physique is closely associated with severe pathology, particularly in the context of cancer. Consequently, participants’ emaciated physique attracted constant scrutiny and triggered disease attributions from both themselves and observers. This attention, even when benevolent, constituted a form of “social gaze” that repeatedly marked the body as pathological. Crucially, these interactions often manifested in an indirect yet unmistakable manner, compelling patients to continually manage, explain, or conceal their illness history in social settings. This constant negotiation hindered their ability to relinquish the social identity of the “cancer patient.”

*"People take one look and ask why I'm not eating properly; some even quietly ask my partner if I've fully recovered," P8; "They don’t mention the illness directly, but when they say I look thinner, I know they’re actually inquiring about my condition… Cancer is, after all, a terrible thing. Others worry that mentioning it might upset me, yet they mean well. So I simply go along with them, pretending I’m not aware of the matter (referring to the illness)," P14.*

## 4 Discussion

This study adopted Interpretative phenomenological analysis (IPA) as its methodological framework. Drawing on in-depth interviews with 16 participates, the research identified core dilemmas rooted in physiological constraints, psychological conflicts, and social pressures associated with nutritional management following esophagectomy. Specifically, a conflict emerged between the objective reality of postoperative physiological limitations and the intense subjective desire to reclaim preoperative normalcy-a tension patients attempted to navigate through active behavioral regulation. Maladaptation to gastrointestinal symptoms precipitated distress stemming from a perceived loss of control, somatic anxiety, and self-doubt. In social settings, patients engaged in self-imposed marginalization to prevent dietary deviations from becoming a focal point of scrutiny. Furthermore, a complex dilemma arose in reconciling familial expectations with individual physiological capacities, while unintentional weight loss served as a persistent, visible manifestation of the disease.

This study found that within symbolic contexts marking the return to a “healthy” state, such as hospital discharge, patients often interpret symptoms as indicators of physical dysfunction and express a strong urgency for their resolution. For example, one participant queried, “How am I still experiencing belching after 4 months?”, reflecting frustration that symptoms persisted beyond the anticipated recovery timeline. This finding aligns with the illness adaptation theory, which posits that while the patient role is socially sanctioned during hospitalization, both patients and their social environments expect a return to normal roles post-discharge, thereby reducing tolerance for residual symptoms (22). Similarly, Watts et al. (23) described the phenomenon wherein cancer patients anticipate a return to everyday life following treatment, yet persistent physical sequelae leave them in a liminal space between health and illness. During this transition period, patients exhibit heightened sensitivity to somatic sensations. Furthermore, the Theory of Symptom Self-Management in Adult Cancer Patients suggests that discrepancies between current functional status and personal or social expectations amplify the patient’s perception of symptom severity (24). This study revealed that such discrepancies precipitated cognitive conflict, motivating patients to actively analyze and interpret gastrointestinal (GI) symptoms while employing self-regulatory strategies-a process detailed in the sub-theme “Dynamic Testing of Dietary and Physiological Boundaries.” Among the side effects of cancer treatment, GI symptoms are the most prevalent and exert the most profound impact on quality of life (20). This coping process aligns with the Common-Sense Self-Regulation Model (CS-SRM), which posits that patients guide their coping strategies based on personal perceptions of the disease and symptoms, including etiology, consequences, and controllability. While rational symptom attribution and active behavioral adjustment can enhance a patients’ sense of control and mitigate anxiety (25). GI symptoms are multifactorial, potentially stemming from physiological impairments, dietary factors, medication side effects, or stress. Health professionals have sometimes not been able to appreciate the impact that treatment-induced side effects can have on the patient and their environment (25). Consequently, facing limited knowledge and professional support, patients encounter significant challenges in accurately attributing the causes of their symptoms. Our study found that patients often experience frustration and learned helplessness when repeated attempts to alleviate symptoms yield no meaningful relief. This psychological state is defined as a sense of powerlessness regarding outcome control that evolves following repeated failures (26). Similarly, research on lung cancer patients demonstrated that frequent experiences of uncontrollable symptoms and stress often fostered a sense of coping powerlessness, characterized by helplessness and self-abandonment when behavioral efforts fail to produce expected outcomes (27). High levels of learned helplessness are strongly associated with reduced self-management capacity; patients subsequently lose confidence in their ability to control and are more likely to abandon coping efforts due to negative expectations. They are more likely to abandon efforts (28). Furthermore, our study revealed that while the discrepancy between current status and expectations initially motivated proactive management, the inability to achieve controlled patients to attribute uncontrollable symptoms to personal inadequacy, a pattern that reinforced learned helplessness. Although these symptoms typically resolve within 3 to 6 months post-surgery, these findings highlighted the importance of helping patients and families establish realistic rehabilitation expectations and facilitating accurate symptom cognition in the early post-operative phase.

This study found that post-esophagectomy patients actively modify their dietary preferences and habits to mitigate perceived risk of recurrence. Food choices are influenced by a multitude of individual, cultural dimension, social and environmental factors (29). In Western culture, cancer patients often believe that positive dietary choices improve overall health, which may indirectly reduce recurrence risk, rather than acting as a direct preventative measure (30, 31). For these patients, the naturalness of food is often a primary motivator (30). However, within the context of Chinese culture and traditional Chinese medicine, the consumption of specific items, such as seafood, spicy food, *fawu*, and the *yin-yang* dietary balance, are believed to be associated with an increased risk of cancer recurrence (32, 33). Accordingly, patients with strong TCM beliefs tend to reduce their consumption of alcohol, seafood, tofu, milk and spicy food (34). The present study further reported that adherence to these dietary restrictions provides patients with a sense of reassurance and agency, reinforcing the belief that they are “actively preventing cancer.” While such modifications can serve as components of a healthy lifestyle and enhance cancer patients’ sense of control over disease outcomes (35), excessive dietary vigilance can have negative consequences. Consistent with our findings, Simons et al. (36) reported that such vigilance diminishes food-related quality of life. Therefore, while encouraging healthy eating habits, healthcare professionals should assist patients in striking a balance that meets nutritional requirements without sacrificing the pleasure and diversity of eating.

In family or social dining settings, visible postoperative dietary modifications often reinforce the “cancer patient” identity. This phenomenon is captured in the sub-theme “Self-Imposed Marginalization at Shared Meals,” where participants reported avoiding communal eating to escape scrutiny. Similarly, Sjeltoft et al. (37) reported that post-surgical changes cause esophageal cancer patients to feel distinct in social settings. Furthermore, conflicts regarding food intake often arise between patients and their families. Hopkinson et al. (38) described similar conflicts in advanced cancer care, attributing them to pressure from caregivers who, driven by anxiety over the patient’s weight loss, urge increased consumption. These dynamic precipitates mealtime distress for patients while simultaneously heightening caregiver anxiety. However, the present study offered a novel insight: shared rehabilitation expectations can create a dilemma where patients struggle to balance personal autonomy with family obligations, resulting in feelings of powerlessness and internal conflict. Therefore, dietary interventions for this population should address the broader family system. Clinical Recommendations include promoting normalized mealtime environments, facilitating the open expression of emotions and needs, and developing collaborative dietary management plans.

Significant weight loss is a prevalent sequel of esophagectomy. A systematic review reported that over half of these patients remain more than 10% below their pre-operative weight 1-year post-surgery; even 3 years later, the average weight remains approximately 14% below baseline (39). Unintentional or perceived weight loss is associated with compromised psychosocial health, including reduced personal control, diminished self-esteem, and strained interpersonal relationships (40, 41). Conversely, interventions providing positive reinforcement regarding weight status have been shown to enhance weight satisfaction and quality of life (42). This study indicated that patients perceive weight recovery as a critical indicator of a “normal” functioning body. When physiological reality contradicted this expectation, they experience profound loss and confusion. They often struggled to accept their altered physiological function, attributing the inability to gain weight to personal inadequacy, a sentiment captured by the query, “What am I doing wrong?” This response reflects an internal attribution bias and shame during rehabilitation, as patients interpret largely irreversible physiological changes as failures of will or behavioral compliance (43). The phenomenon of “eating without weight gain” is frequently interpreted as a sign of persistent disease, exacerbating anxiety and uncertainty regarding cancer recurrence. Physical emaciation also served as a visible stigma of illness, attracting recurrent scrutiny in social settings. As one participant lamented, “I’ve become so thin that I look ill,” while another described wearing multiple layers “to appear a bit heavier.” These experiences underscore how visible weight loss reinforces an “illness identity,” disrupts body image, and precipitates social withdrawal, ultimately contributing to adverse mental health outcomes such as depression, low self-esteem, and diminished wellbeing (44). While body image is recognized as a critical determinant of quality of life in cancer survivorship, existing research has predominantly focused on breast, prostate, colorectal, and head and neck cancers, as well as adolescent populations and cachexia in advanced disease (45, 46). Rhoten (47) delineated three core attributes of body image disturbance in cancer patients: dissatisfaction with perceived appearance changes, functional decline, and the resultant psychological distress. Among upper gastrointestinal cancer survivors, these attributes manifest uniquely. Unlike the localized anatomical alterations typical of breast or colorectal cancer (48, 49), the distress in this population stems from systemic and sustained emaciation—a whole-body transformation perceived as a visible stigma of severe pathology. Consequently, this study highlighted that weight loss and altered body image were central drivers of identity conflict, social reintegration challenges, and psychological distress.

### 4.1 Strengths and limitations of the study

This study employed IPAs to explore the patients’ subjective construction of nutritional meanings, revealing that nutrition management is not merely a physiological process but also a multidimensional adaptive process encompassing cognition, emotion, family dynamics, and social identity. These insights address gaps in the quantitative literature by elucidating the specific mechanisms through which nutritional challenges influence mental health. However, as a qualitative study, this research focuses on the nature of the association between nutritional management and mental health rather than establishing statistical causality. Consequently, it does not quantify the magnitude of relationships among nutritional status, psychological adaptation, and social functioning. This study relied primarily on patient perspectives, which may not fully capture the complexity of family dynamics. The exclusion of family members limited the ability to corroborate findings regarding dietary expectations and relational tensions. Consequently, results concerning “family dietary contexts” and “reconciling family expectations” should be interpreted as reflecting the patients’ subjective perceptions rather than reciprocal dyadic or systemic family processes. Future research would benefit from a multi-perspective design to address this limitation. Additionally, participants were recruited exclusively from hospitals within a single region, potentially introducing geographical and population biases that limit the generalizability of the findings. Furthermore, the study population consisted entirely of older adults (aged 58–75 years; mean = 64.6). Although this cohort is representative of the typical demographic for esophageal cancer, the results may not be fully applicable to younger survivors, who likely encounter distinct psychosocial challenges related to employment, familial responsibilities, and long-term survivorship.

### 4.2 Future research direction

Future research could expand the study population to include a broader range of upper gastrointestinal (GI) cancers, such as gastric and pancreatic cancer, to compare how the interplay between nutrition and mental health varies across different postoperative cohorts. Utilizing mixed-methods approaches within multicenter, large-sample epidemiological surveys would allow for the inclusion of patients from diverse regions, demographics, and treatment stages, thereby facilitating a more comprehensive evaluation of the mechanisms through which nutritional management impacts mental health. Longitudinal studies are also needed to track the temporal trajectories of nutritional status and psychological wellbeing, helping to identify critical turning points and influencing factors that can inform precision interventions. Additionally, integrated nutritional-psychological intervention programs, such as family-based nutritional education and cognitive behavioral therapy (CBT) support groups, should be developed and rigorously evaluated to address nutrition-related psychological distress. Finally, the role of digital health tools in supporting postoperative adaptation warrants further exploration. Investigating the efficacy and acceptability of mobile applications for dietary monitoring and remote consultations could provide scalable, technology-enhanced solutions to improve long-term self-management and psychosocial outcomes in this population.

## 5 Conclusion

This study investigated the interplay between nutritional factors, mental health, and identity adaptation in cancer patients following esophagectomy. The findings demonstrated that challenges in cognitive and behavioral adaptation to gastrointestinal symptoms, coupled with role restructuring and emotional negotiation within family dietary contexts, contribute to the development of adverse psychological outcomes. Additionally, the struggle for weight restoration emerged as a significant compounding factor. From the perspective of self-cognition, this study provides insights into how nutritional challenges shape the mental health of cancer patients within the Chinese cultural context. Based on these findings, we underscored the necessity of patient-centered nutritional interventions. These strategies should prioritize alleviating symptom-related anxiety, facilitating reintegration into family dietary routines, and reconciling healthy eating guidelines with individual dietary preferences.

## Data Availability

The original contributions presented in the study are included in the article/Supplementary material, further inquiries can be directed to the corresponding author.
