# Supplementary figures and images for: The distress of psychological adaptation in nutritional management among people after esophagectomy: an interpretative phenomenological study
Source: Front Nutr. 2026 Feb 27;13:1720415. doi: 10.3389/fnut.2026.1720415 (PMC12982023; doi:10.3389/fnut.2026.1720415)

Supplementary Image 1: Coding tree of themes and sub-themes.

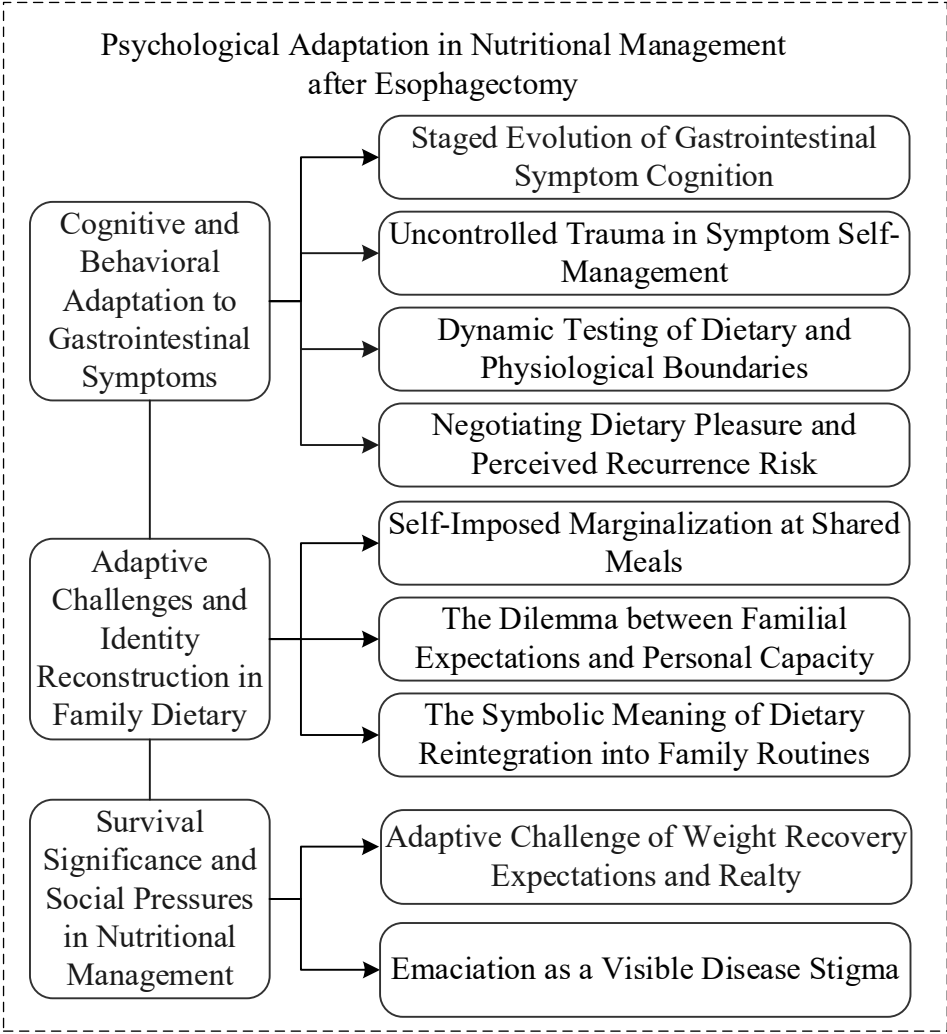

Supplement: Supplementary file 2 [file Image_1.pdf]
